# Detection of Escherichia coli in Food Samples Using Culture and Polymerase Chain Reaction Methods

**DOI:** 10.7759/cureus.32808

**Published:** 2022-12-21

**Authors:** Sumyya Hariri

**Affiliations:** 1 Department of Microbiology, Umm Al-Qura University, Mecca, SAU

**Keywords:** food poisoning, food pathogens, culture, enrichment, e. coli, pcr, food

## Abstract

Methods for the rapid detection of *Escherichia coli* and its related toxins are key to minimizing the risk of exposure to foodborne pathogens. The present study aimed to detect *E. coli* in food specimens using culture and polymerase chain reaction (PCR) techniques. One hundred and fifty samples from different types of food, comprising beef (n=60), chicken (n=72), and fish (n=18), were analyzed for the identification of *E. coli *by conventional and PCR methods. The results showed that out of 150 food samples, 44 (29.3%) were positive by culture, and 50 (33.3%) were positive by PCR. Significant differences were detected between sample types with culture (p-value < 0.005). When culture was considered the gold standard, the sensitivity of PCR was 100%, while the specificity was 94.34%. The six-hour pre-enrichment and PCR analysis are reliable in fast detection of *E. coli* in food samples. Hence, the identification of food pathogens using molecular-based methods would become more useful in routine diagnostic laboratories

## Introduction

Food-borne illnesses are now becoming a serious threat to public health. These illnesses are caused by microorganisms (bacteria, viruses, fungi, and other parasites) or by chemical substances that contaminate food or water and cause more than 2500 diseases [1]. Worldwide, it has been reported that 600 million people get sick with food-borne illnesses, and 420,000 die each year from food poisoning [1, 2]. *Escherichia coli* is a large group of gram-negative bacteria and, in most cases, is harmless. *E. coli* is also one of the most important microorganisms used for the monitoring of water and food safety. Some strains of *E. coli* (O157:H7 (STEC)) are commonly associated with food poisoning outbreaks [3]. Over 700 strains or serotypes of *E. coli* exist in nature, water, and foods. *E. coli *possesses quite a few virulence factors encoded on mobile genetic elements and/or plasmids or localized in pathogenicity islands. These virulence factors are expressed as endotoxins, exotoxins, adhesion, invasion, or iron acquisition factors.

Health and human concerns are concentrated on the few pathogenic strains, such as strain O157:H7 (the cause of bloody diarrhoea from eating undercooked or raw meat), non-O157:O7 strains (O26, O145, O111, O121, and O45), and Shiga toxin-producing *E. coli* [4]. Methods for the rapid detection of *E. coli* and its related toxins are key to minimizing the risk of foodborne pathogens. Consequently, many detection methods have been developed, particularly to detect *E. coli* in water and foods. Bacterial isolation and identification by culturing are the standard methods for the detection of foodborne pathogens in addition to Gram staining, bacterial count, and biochemical testing [5]. These methods are labour-intensive, slow, and time-consuming (2-10 days for initial results up to confirmation). Therefore, accurate, fast, and low-cost detection methods are needed to assess the microbiological status of food [6].

To minimize the risk of *E. coli*, many efforts including improvement of diagnostic methods should be spent especially in food establishments. Other techniques, such as adenosine triphosphate (ATP) and optical methods, have also been used for the rapid detection of *E. coli*. However, such methods involve high costs, sensitive chemicals, and periodic maintenance [7-10]. Some recent rapid detection technologies for pathogenic bacteria include immunoassays, nucleic acid-based assays such as polymerase chain reaction (PCR), DNA microarrays, and antibody-based assays [11-14]. It has been shown that these methods have a short operating time, and their detection time is also low (2-24 hours) [11-14]. However, some of these methods still have low sensitivity and selectivity and, hence, need improvements in accuracy to be of any practical use. The present study aimed to detect *E. coli* in food samples using culture and PCR techniques.

## Materials and methods

One hundred fifty samples from different types of food were purchased from hotels, restaurants, and cafeterias in Mecca city, Saudi Arabia. The selected food samples, consisting of beef (n=60), chicken (n=72), and fish (n=18), were labelled, recorded, and immediately transported in an icebox with freeze packs under sterile conditions to the laboratory of the Department of Environment and Health Research, the Custodian of the Two Holy Mosque Institute for Hajj and Umrah Research, and analysed within two hours hours of collection. If delayed, the samples were refrigerated at 0-4 °C for no more than 24 hours after collection. The isolates were grown at 37 °C and 150 rpm for 10 hours. Two types of growth media were used: (i) Luria-Bertani (LB) broth for general purposes and (ii) Tryptone Bile X-glucuronide (TBX) agar medium, a selective medium for the enumeration and differentiation of *E. coli* from other coliforms [15]. The two growth media were used for the identification of *E. coli* by conventional (culture and biochemical testing) and PCR methods.

Standard microbiological methods

Isolation and identification of *E. coli* were performed by standard microbiological methods. The homogenized samples were transferred into the nutrient broth (5 ml/test tube) and MacConkey agar (Oxoid Limited, Hampshire, United Kingdom). Any grown colony was picked, Gram stained, subjected to different biochemical tests (sugar fermentation, indole production, methyl-red, Voges-Proskauer, and citrate utilization tests), and then subcultured onto Eosin Methylene Blue (EMB) agar.

PCR after pre-enrichment

The pre-enrichment of samples was performed according to the method described by Medici et al. [16] with some modifications. Approximately 25 g samples were homogenized with 225 mL of buffered peptone water (BPW) medium (Oxoid, CM0509) and then divided into two aliquots. While one aliquot was incubated at 37 °C for 24 hours, the other aliquot was subjected to pre-enrichment culture for six hours. The first aliquot was used for DNA extraction by enzyme and freeze/thaw methods, and the second aliquot was used to confirm the presence of *E. coli *by standard culture methods, followed by biochemical and serological confirmatory tests. The DNA was extracted from the first aliquots by the Boil Lysis Method following Ahmed and Dablool [17]. Briefly, the aliquot was boiled at 100 °C for 10 minutes. Insoluble material was discarded through centrifugation for two minutes, and the supernatant was used as a template in the PCR. A PCR mixture (50 µL) containing one 100 pmol of each primer, 3 µL of DNA template, and a 25 µL solution of Taq PCR Master Mix polymerase (Promega Corporation, Madison, Wisconsin, United States) was used to conduct the PCR. Using Mastercycler® personal PCR equipment (Eppendorf, Hamburg, Germany), DNA was amplified under the following conditions: heat denaturation at 94 °C for three minutes, followed by 30 amplification cycles (one minute at 94 °C, 65 seconds at 62 °C, and 90 seconds at 72 °C), and an elongation step of seven minutes at 72 °C. The primers used were Afa FP (5' GCT GGG CAG CAAACT GAT AAC TCT C 3') and Afa RP (5' CAT CAA GCT GTT TGTTCG TCC GCC G 3'), which amplify a 480 -bp fragment within the conserved Afa gene sequence of pathogenic *E. coli *[18] using Mastercycler. The products were electrophoresed in a 1.5% agarose gel for one hour at 120 V, stained with ethidium bromide and visualized under ultraviolet light using a gel documentation system via an ultraviolet transilluminator (UVP BioDoc-It® Imaging System; Ultra-Violet Products Ltd, Cambridge, United Kingdom).

Data analysis

Data were analysed, and statistical analyses were made by using the chi-square method using IBM SPSS Statistics for Windows, Version 25.0 (Released 2017; IBM Corp., Armonk, New York, United States). A p < 0.05 was considered statistically significant.

## Results

**Figure 1:**
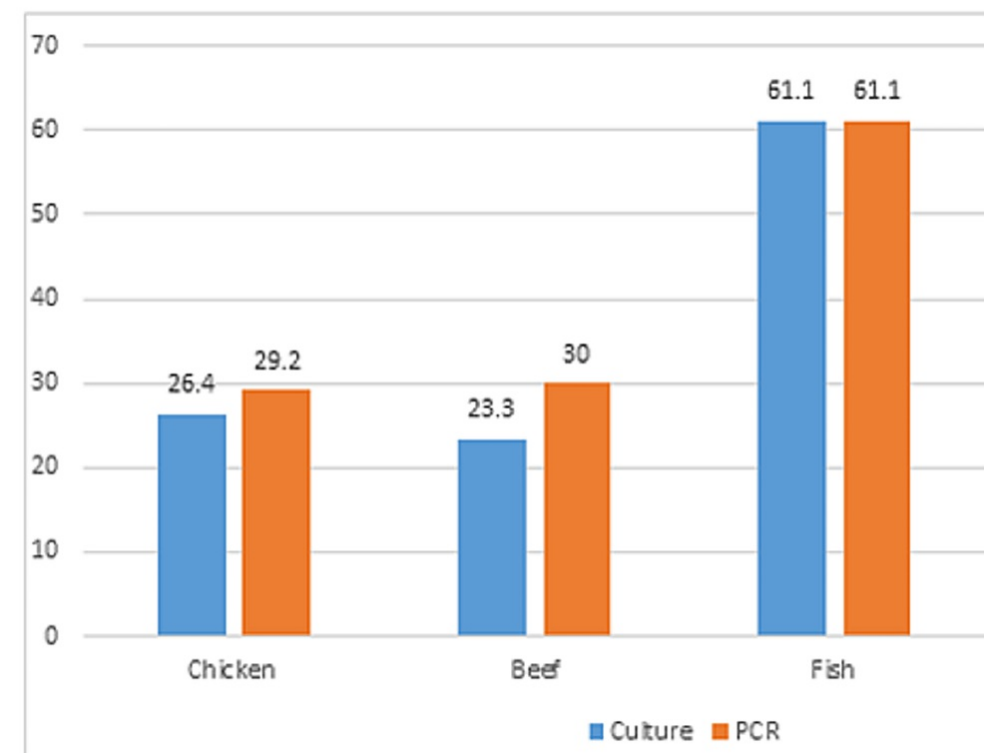
Distribution of Escherichia coli isolates among food samples PCR: polymerase chain reaction

**Figure 2:**
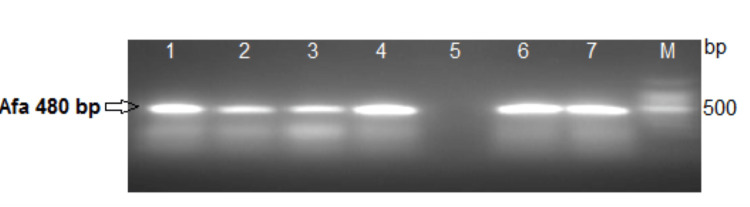
PCR analysis of Escherichia coli isolates among food samples Lane 1: Control positive; Lanes 2, 3, 4, 6 and 7: positive Afa gene of *Escherichia coli* strains (480 bp); Lane 5: Control negative; Lane M: 100-bp DNA ladder. PCR: polymerase chain reaction

**Figure 3:**
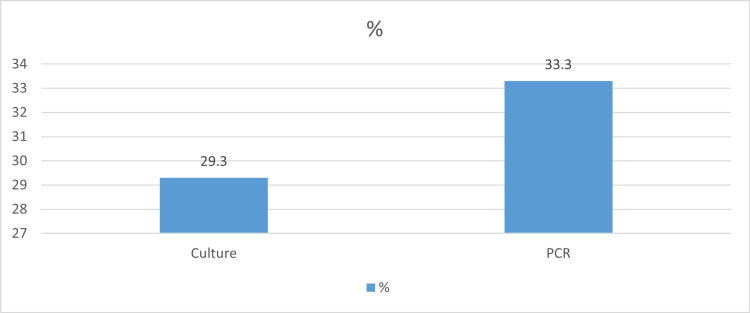
Frequencies of positive samples for Escherichia coli by using culture and polymerase chain reaction

Out of 150 food samples, 44 (29.3%) were positive by culture method and 50 (33.3%) were positive by PCR assay, as shown in Table 1 and Figure 1. Figure 2 shows the results of the food analysis for *E. coli*. PCR (Figure 3) showed the highest rate of *E. coli* in fish samples (61.1%), followed by beef (30.0%) and chicken (29.2%). Culture showed the highest rate of *E. coli *in fish samples (61.1%), followed by chicken (26.4%) and beef (23.3%).

**Table 1:** Frequencies of positive samples for Escherichia coli in different samples PCR: polymerase chain reaction

Result	Chicken (n=72)	Beef (n=60)	Fish (n=18)	Total	p-value
Culture	19 (26.4%)	14 (23.3%)	11 (61.1%)	44 (29.3%)	0.006
PCR	21 (29.2%)	18 (30.0%)	11 (61.1%)	50 (33.3%)	0.29

Significant differences were detected between sample types with culture (p-value < 0.05), as shown in Table 1. When culture was considered the gold standard, the sensitivity of PCR was 100%, while the specificity was 94.34%, with a positive predictive value (PPV) of 89.82% and a negative predictive value (NPV) of 100% (Table 2).

**Table 2:** Sensitivity and specificity of PCR method when culture is gold standard PCR: polymerase chain reaction; PPV: positive predictive value; NPV: negative predictive value

	PCR
Sensitivity	100%
Specificity	94.34%
PPV	89.82%
NPV	100%

## Discussion

Food hygiene, particularly microbiological safety, is an important concern in the food industry, especially during mass gatherings. *Listeria* *monocytogenes*, *E. coli*, *Staphylococcus aureus*, *Salmonella enterica*, and *Bacillus cereus* are the common food-borne pathogenic bacteria that cause the majority of food-borne disease outbreaks [19]. Methods for the rapid detection of food-borne infections are becoming increasingly crucial in many food analyses, as the traditional approaches are time-consuming and labour-intensive.

*E. coli* detection in foods is one of the most useful hygienic criteria because of the involvement of the bacterium in causing diseases in addition to its use as an indicator of the presence of other pathogens in foods [20]. The present study aimed to detect *E. coli* using culture and PCR techniques in different types of food (chicken, beef, and fish) purchased from catering establishments in Mecca city during mass gathering events. The results of the present study showed that 33.3% of the food samples were positive by PCR, while 29.3% of them were positive by culture. Detection of *E. coli* by culture followed by standard biochemical identification remains the method of choice, especially during outbreaks due to the potential need to compare the results with other typing methods.

Culture results showed the highest rate of *E. coli* in fish samples (61.1%), followed by chicken (26.4%) and beef (23.3%). However, culture methods may be laborious, have low sensitivity, be time-consuming and require a long time from two days to more than a week for pathogen confirmation. More rapid, sensitive, and specific methods, such as nucleic acid-based methods, have low numbers of false-positive results and no false-negative results and are thus more reliable to detect and identify *E. coli *in food samples.

Previously, many PCR-based detection methods for the determination of *E. coli *in food specimens have been developed that target *E. coli* genes [21]. By using PCR, the present study showed that the highest rate of *E. coli* was detected more in fish samples, followed by beef and chicken. In a previous study, all *E. coli *in food specimens were detected after an enrichment period of six hours [22]. In another study from India, 50% of the food samples tested were considered positive for *E. coli*, where the highest level of incidence of *E. coli* was found in beef samples (91%) [23]. In Mexico, 44% of the food samples were positive for *E. coli* [24]. The PCR method was tested by Nguyen et al. on three different types of samples, including chicken meat, and it was shown that after 12 hours of enrichment against foodborne pathogens such as *E. coli*, the lowest detection level of 10 CFU/mL was achieved [25]. Therefore, the three main food-borne pathogens may be reliably and effectively detected using this multiplex PCR assay in conjunction with a pre-enrichment method.

When culture was considered the gold standard, the sensitivity of PCR was 100%, while the specificity was 94.34%. Comparatively, in a study by Zhang et al., the maximum sensitivity of 100 CFU/mL for four pathogens, including *E. coli*, was obtained after 24 hours of enrichment from eight experimentally contaminated food samples [26]. The importance of the present study may be seen in the mass gatherings as the presence of a large number of people in overcrowded conditions considerably increases the risk of occurrence of food poisoning and gastrointestinal diseases caused by uncooked food, so that earlier PCR assays also yielded the simultaneous detection of food-borne pathogens, including *E. coli*, at low concentrations [27].

## Conclusions

The PCR achieved was successful in detecting the presence of *E. coli* in food specimens (33.3%) with 100% sensitivity and 94.34% specificity, while the culture method detected *E. coli* in 29.3% of the specimens. The six-hour pre-enrichment of samples and PCR analysis using Afa gene-specific primers can reliably and effectively detect *E. coli* rapidly in food samples. Hence, the identification of food pathogens using molecular-based methods will become more useful in routine diagnostic laboratories, especially during mass gatherings where the risk of occurrence of food poisoning is high.
